# Laser capture microdissection (LCM) and whole genome amplification (WGA) of DNA from normal breast tissue --- optimization for genome wide array analyses

**DOI:** 10.1186/1756-0500-4-69

**Published:** 2011-03-18

**Authors:** Kristina E Aaltonen, Anna Ebbesson, Caroline Wigerup, Ingrid Hedenfalk

**Affiliations:** 1 Department of Oncology, Clinical Sciences, Lund, Lund University, Barngatan 2B, SE-221 85 Lund, Sweden; 2 Department of Laboratory Medicine, Center for Molecular Pathology, Lund University, University Hospital MAS, SE-20502 Malmö, Sweden

## Abstract

**Background:**

Laser capture microdissection (LCM) can be applied to tissues where cells of interest are distinguishable from surrounding cell populations. Here, we have optimized LCM for fresh frozen normal breast tissue where large amounts of fat can cause problems during microdissection. Since the amount of DNA needed for genome wide analyses, such as single nucleotide polymorphism (SNP) arrays, is often greater than what can be obtained from the dissected tissue, we have compared three different whole genome amplification (WGA) kits for amplification of DNA from LCM material. In addition, the genome wide profiling methods commonly used today require extremely high DNA quality compared to PCR based techniques and DNA quality is thus critical for successful downstream analyses.

**Findings:**

We found that by using FrameSlides without glass backing for LCM and treating the slides with acetone after staining, the problems caused by excessive fat could be significantly decreased. The amount of DNA obtained after extraction from LCM tissue was not sufficient for direct SNP array analysis in our material. However, the two WGA kits based on Phi29 polymerase technology (Repli-g^® ^(Qiagen) and GenomiPhi (GE Healthcare)) gave relatively long amplification products, and amplified DNA from Repli-g^® ^gave call rates in the subsequent SNP analysis close to those from non-amplified DNA. Furthermore, the quality of the input DNA for WGA was found to be essential for successful SNP array results and initial DNA fragmentation problems could be reduced by switching from a regular halogen lamp to a VIS-LED lamp during LCM.

**Conclusions:**

LCM must be optimized to work satisfactorily in difficult tissues. We describe a work flow for fresh frozen normal breast tissue where fat is inclined to cause problems if sample treatment is not adapted to this tissue. We also show that the Phi29-based Repli-g^® ^WGA kit (Qiagen) is a feasible approach to amplify DNA of high quality prior to genome wide analyses such as SNP profiling.

## Findings

### Background

Laser capture microdissection (LCM) is a widely used method for isolation of defined cell populations from heterogeneous tissue sections. The method allows selection of unmixed starting material for DNA, RNA or protein extraction for further downstream molecular analyses [1-3]. However, LCM needs to be optimized depending on tissue type, for example normal breast tissue contains more lipids than breast tumor tissue. The PALM MicroBeam system (Carl Zeiss MicroImaging, Jena, Germany) is one of several commercially available LCM systems. This system utilizes a UV laser to cut around the selected cells and a pulse from the same laser to catapult the selected specimen into a collection device, e.g. an AdhesiveCap microcentrifuge tube (Carl Zeiss MicroImaging). In order to catapult larger tissue structures with a single laser pulse, slides with thin polyethylene membranes are used. There are two types of slides available; MembraneSlides, which are regular glass slides covered by the membrane and FrameSlides where the membrane is only supported by a metal frame.

The amount of DNA yielded by LCM can be sufficient for PCR based analyses, but is often insufficient for genome-wide applications such as high density single nucleotide polymorphism (SNP) genotyping arrays. Whole genome amplification (WGA) provides a possibility to amplify a small amount of high quality DNA and there are several WGA methods available. Many WGA kits on the market today employ the multiple displacement amplification (MDA) technology (e.g. GenomiPhi (GE Healthcare Life Sciences, Uppsala, Sweden) and Repli-g^® ^(Qiagen, Hilden, Germany)). This Phi29 DNA polymerase-based technique has in many studies been found to provide the most balanced genome amplification to date [4-7]. The concordance between non-amplified and MDA amplified DNA in SNP arrays has been found to be higher than 98% [8-10] and a majority of studies report MDA WGA to be the method of choice for SNP array analyses [5,11,12]. However, the key to accurate WGA is high quality input DNA and the DNA quality after LCM is difficult to investigate since the small amount of DNA obtained after microdissection restricts the methods available for quality assurance.

In this study, we have developed a protocol for LCM of fresh frozen normal breast tissue to enable microdissection of this challenging, fat rich tissue. We have also tested three different, commercially available, WGA kits to obtain sufficient amounts of high quality DNA for high-density SNP array analysis.

### Tissue preparation and laser capture microdissection (LCM)

**Figure 1:**
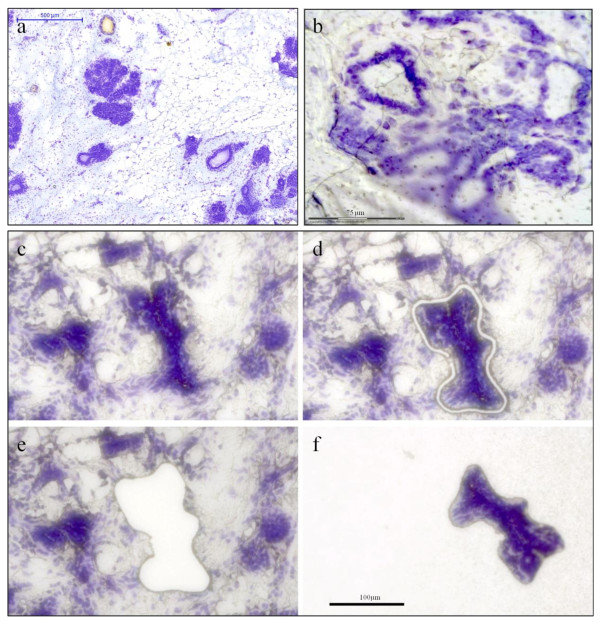
**Cresyl Violet stained breast tissue**. a) Low magnification overview, scanned with a MiraxViewer slide scanner; b) Illustration of how lipids from normal breast tissue can collect between the membrane and the slide on MembraneSlides and cause technical difficulties; c) Overview of the LCM area; d) Cut line around structure of interest; e) Area after catapulting; f) Catapulted piece collected in the cap.

**Figure 2:**
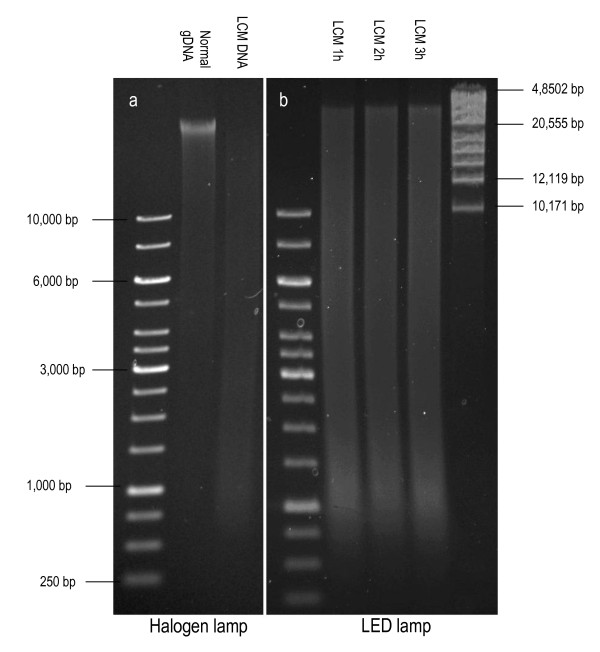
**Visualization of DNA on 0.5% agarose gels stained with EtBr after a) dissection under standard halogen lamp (Lane 1 = good quality genomic DNA (gDNA) from blood, Lane 2 = fragmented DNA after LCM for approximately 2 hours) and; b) after 1, 2 or 3 hours of LCM under a VIS-LED lamp **.

Samples of histologically normal breast tissue were collected from women who had undergone a prophylactic mastectomy due to increased risk of developing breast cancer (see Rennstam *et al *[13] for details). Informed consent forms were signed by all women included in the study and the study was approved by the ethics committee at Lund University, Sweden. The tissue was snap frozen and kept at -80°C. On the day of LCM, 16 μm sections were cut at -20°C in a cryostat and placed on MembraneSlides (Carl Zeiss MicroImaging). Cresyl violet staining performed according to a standard protocol from Zeiss Labs, Munich, Germany (http://www.zeiss.de/ microdissection) gave a distinct nuclear staining that made it easy to identify breast ducts and lobules (Figure 1a). This protocol included fixation in 70% ethanol (EtOH) for 2 minutes followed by staining with a Cresyl Violet (CV) solution (1% w/v in 50% EtOH) for 45 seconds before washing and dehydration, first in 70% EtOH and then in 100% EtOH, 10 dips in each. When using MembraneSlides, "bubbles" formed underneath the tissue (Figure 1b) and caused focusing problems, making cutting virtually impossible. Assisted by Zeiss Labs, Munich, Germany (personal communication) we realized that the bubbles were caused by lipids leaking through the membrane. By switching from MembraneSlides to FrameSlides (Carl Zeiss MicroImaging) this problem was solved. Also, by adding a 20 second rinse in acetone as the last step in the staining protocol, more fat was dissolved and the catapulting improved greatly. It should be noted, however, that acetone weakens the adhesion of the tissue to the membrane and rinses longer than 20 seconds should be avoided. Acetone was chosen instead of xylene, since PALM Protocols from Zeiss Labs advise against using xylene, due to increased tissue brittleness as well as decreased adhesion to the membrane. An example of a typical area for LCM is shown in Figure 1c-f. Note the bridge, which is left uncut to prevent the tissue from dropping down (Figure 1d). This bridge is also used as the point of catapulting. Unexpectedly, LCM was found to negatively affect the quality of the resulting genomic DNA (gDNA) (Figure 2a), thereby influencing subsequent SNP array analyses. The problem could be solved by switching from the standard halogen lamp to a VIS-LED lamp which generates noticeably less heat in the LCM microscope. The time spent on LCM (1, 2 or 3 hours) did not visibly affect the DNA quality (Figure 2b).

### DNA extraction and whole genome amplification (WGA)

DNA was extracted directly after dissection using the QIAmp DNA Micro kit (Qiagen) according to their specific protocol for extraction of genomic DNA from LCM tissues. A few modifications were made to the protocol: Buffer ATL and proteinase K were mixed before addition to the AdhesiveCap and no vortexing of the tubes was performed before 4 hours incubation at 56ºC in an up-side-down position. DNA concentrations were measured on the Qubit™ quantification platform using the Quant-iT™ high sensitivity assay for double stranded DNA (dsDNA) (Invitrogen, Carlsbad, CA). The DNA yield was approximately 30 ng DNA per mm^2 ^of microdissected tissue.

**Figure 3:**
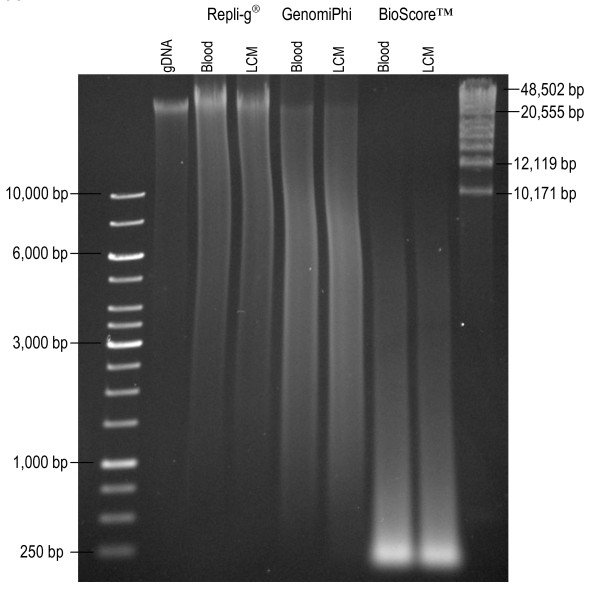
**Amplification products from the three different WGA kits on a 0.5% agarose gel stained with EtBr **.

For WGA, three different methods were tested on a pool of high quality DNA extracted from blood as well as on DNA from LCM tissue. Different concentrations of DNA were tested as input from LCM samples (Table 1). For all three kits the protocols provided by the manufacturers were carefully followed and the concentration of dsDNA after WGA was determined using the Quant-iT™ broad range assay (Invitrogen). The DNA input and average output are summarized in Table 1. The size and quality of the amplified fragments were examined on a 0.5% agarose gel stained with ethidium bromide. Between 150 and 200 ng DNA were loaded in each lane. The first WGA kit, Repli-g^® ^from Qiagen uses Phi29 DNA polymerase to amplify low quantities of DNA by MDA. The length of the fragments was longer than 10 kb, suggesting high DNA quality suitable for SNP arrays (Figure 3). There is also a user-developed protocol for direct WGA of microdissected tissue available at Qiagen's homepage (http://www.qiagen.com). This protocol was tested with an approximately 1 mm^2 ^input area of LCM tissue and the amplification products were found to be surprisingly long (data not shown). However, SNP array call rates clearly discourage this method of amplification before SNP array studies (Table 2). The second kit, GenomiPhi V2 DNA amplification kit (GE Healthcare) is also a Phi29 based MDA kit and the quality of the amplification product was relatively high, with many fragments longer than 3 kb (Figure 3). Finally, the BioScore™ FFPE Screening and Amplification kit (Enzo Life Sciences), which is developed especially for formalin-fixed paraffin-embedded tissue that is normally difficult to amplify due to fragmented and cross-linked DNA, was tested. It is a non-MDA based kit with a proprietary mechanism that is not disclosed to the customer (Enzo Life Sciences, personal communication). Here, this protocol gave the highest DNA yield but it also required the highest input amounts (Table 1). The DNA quality, with many short fragments, was clearly inferior to the other kits (Figure 3) and it should be noted that the price for this more specialized kit is considerably higher.

**Table 1:** WGA using three different commercial kits on DNA extracted from microdissected tissue and from blood

			**DNA SOURCE**			
			**LCM tissue**		**Blood**	
			
	**Recommended input (μg)**	**Expected output (μg)**	**Input (μg)**	**Output (μg)**	**Input (μg)**	**Output (μg)**
Repli-g^® ^(Qiagen)	>0.010	Approx. 10	0.015-0.030	2.0 (0.5-5.1)	0.013	5.2 (3.9-6.5)
GenomiPhi (GE Healthcare)	>0.010	4-7	0.013-0.050	2.0 (1.1-3.6)	0.013	4.5 (4.2-4.8)
BioScore™ (Enzo Life Sciences)	0.100	>10	0.040-0.100	12.9 (11.9-14.3)	0.100	11.8 (10.3-13.3)

The recommended input and expected output are noted for each kit as well as the results from this experiment. The output from this experiment is given as mean values and the range within brackets.

**Table 2:** Call rates for SNP arrays

	**Blood**	**LCM tissue**
		
Non-amplified	0.997*	0.997**
Repli-g^®^	0.994*	0.987**
Direct ampl. Repli-g^®^		0.755
BioScore™	0.975*	
GenomiPhi	0.956*	

DNA from blood and LCM tissue was amplified with different commercial kits. Stars indicate that input DNA comes from the same pool of DNA extracted from blood (*) or LCM tissue (**), respectively. Call rates for non-amplified DNA and WGA products from Repli-g^® ^(bold) are comparable and suggest that this method is best suited for whole genome amplification before SNP array analysis.

### SNP array

SNP analyses were run on Infinium Human Omni 1 M arrays (Illumina, San Diego, CA) at the Swegene Centre for Integrative Biology at Lund University (SCIBLU) genomics facility (Lund University, Sweden). Data analyses were performed in Illumina's GenomeStudio^® ^Genotyping Module. The SNP array call rates are listed in Table 2 and show that amplification products from both blood and tissue with the Repli-g^® ^kit (Qiagen) give call rates comparable to what was obtained with non-amplified blood and tissue. Non-amplified DNA, however, showed less noise in the SNP profiles. GenomiPhi and BioScore™ amplified DNA from blood caused more noise in the SNP analysis and also displayed clearly lower call rates than non-amplified DNA from blood. These kits were therefore not further applied to DNA from LCM tissue. Interestingly, the direct-amplified (Repli-g^®^, Qiagen) LCM tissue without previous DNA extraction gave extremely poor call rates, and the high noise level suggests that this amplification method is clearly not suited before genome wide analyses.

## Discussion

We have developed a protocol for LCM of fresh frozen normal breast tissue, which is a demanding tissue to work with and we believe the method to be valid for other lipid-rich tissues as well. A switch to FrameSlides and an added acetone wash were necessary for LCM to work adequately. When using FrameSlides, however, it was obvious that some tissue was dropped and could be found on the objective below the stage. This loss, together with other pieces that were not successfully catapulted into the cap, was estimated to approximately 10% and is in line with observations made by other groups [14]. Interestingly, other groups working with breast tissue have not described problems with LCM. However, much of the previous work has been performed on paraffin embedded tissue [15,16] where all lipids have been removed during the embedding process. For frozen sections, most work has been performed on breast tumor tissue [2,17], which contains considerably less adipose cells than normal breast tissue. To our knowledge, in the few studies where normal fresh frozen breast tissue has been studied, LCM systems by Arcturus Engineering have been used [18-22]. Possibly, these systems are less sensitive to contaminating lipids.

We performed a thorough quality control of DNA obtained from LCM tissue after recognizing that a lack of long fragments in the input to WGA did not result in unsuccessful amplification but was only observable as low SNP array call rates. As displayed in the gel image in Figure 2a, the majority of the LCM DNA fragments were shorter than 4 kb. We found that the original halogen lamp in the LCM microscope was causing DNA fragmentation during microdissection, probably due to heat production. After changing to a VIS-LED lamp, clearly higher DNA quality was obtained (Figure 2b). We suspect that poor DNA quality is often a problem in the analysis of DNA obtained from LCM, but that this is rarely discovered since the small amount of DNA limits the options for quality control of long fragment DNA. Most likely, the DNA quality after LCM is sufficient for most PCR-based assays and possibly for other less sensitive analyses. However, we found that for SNP array analyses the quality of the input DNA is crucial for a successful outcome after WGA.

**Figure 4:**
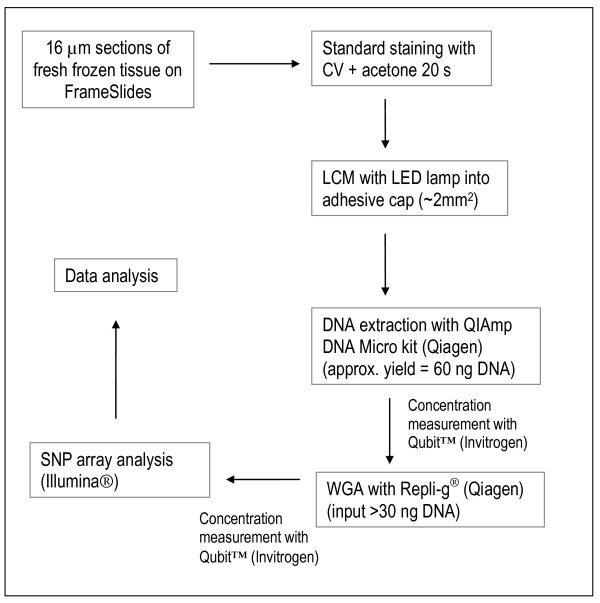
**Flow chart describing our work flow from tissue sectioning of fresh frozen breast tissue to SNP array data analysis**. Details on the different steps are found in the text.

The amplification products after WGA clearly differed in DNA yield and quality (Table 1 and Figure 3). The BioScore™ WGA kit is developed for formalin-fixed, paraffin-embedded tissue and as a consequence, the amplification products are noticeably shorter than from the other two kits (Figure 3). Since our material is fresh-frozen, we had the possibility to use one of the two MDA-based kits that give clearly longer amplification products. According to the literature, Qiagen's Repli-g^® ^kit is often the method of choice for whole genome amplification before SNP array analyses [10,11,23] whereas the GenomiPhi kit (GE Healthcare) is often chosen when working with microdissected tissue [24-27]. We tested both the GenomiPhi and Repli-g^® ^methods and found that the Repli-g^® ^kit gave DNA fragments of approximately the same size as genomic DNA (Figure 3). The GenomiPhi kit also gave products longer than approximately 1 kb and up to more than 10 kb. We did not find a correlation between the amount of starting material in the WGA reactions and the output after amplification. However, the quantity of starting material has previously been shown to affect the level of amplification bias in the product. Arriola *et al. *[28] showed that an input of 0.5-10 ng resulted in a higher amplification bias than when larger amounts of starting DNA were used. It is therefore suggested that the same amount of starting material should be used in both test and reference samples since copy number change biases were found to be non-random [9,28]. Our results also show a difference in call rates between amplified and non-amplified products, thus indicating that the reference and test samples should indeed be treated equally in this respect. However, call rates for MDA products have generally been found to be high and sometimes even comparable with call rates for genomic DNA [9,10]. We found this to be true in Repli-g^® ^amplified DNA (Table 2) and thus suggest that whole genome amplification with this kit is best suited for SNP array analyses. Nevertheless, amplified DNA tends to yield noisier data and adequate normalization and filtering always need to be performed by the user. The flow chart in Figure 4 summarizes the different steps suggested for LCM and WGA before SNP array analysis of DNA from normal breast tissue.

## Competing interests

The authors declare that they have no competing interests.

## Authors' contributions

KEA carried out the WGA studies, participated in LCM and drafted the manuscript. AE carried out sectioning and staining, participated in LCM and drafted the manuscript. CW participated in WGA and LCM. IH conceived of the study and helped to draft the manuscript. All authors read and approved of the final manuscript.
